# Breech delivery at a University Hospital in Tanzania

**DOI:** 10.1186/s12884-016-1136-0

**Published:** 2016-11-08

**Authors:** Ulf Högberg, Catrin Claeson, Lone Krebs, Agneta Skoog Svanberg, Hussein Kidanto

**Affiliations:** 1 Department of Women’s and Children’s Health, Uppsala University, SE-751 85 Uppsala, Sweden; 2 Department of Obstetrics and Gynecology, Karolinska University Hospital, 171 76 Solna, Sweden; 3 Department of Obstetrics and Gynecology, University of Copenhagen, and Holbæk Hospital, Copenhagen, Denmark; 4 Department of Obstetrics and Gynecology, Muhimbili National Hospital, Dar-es-Salaam, Tanzania

**Keywords:** Breech presentation, Caesarean section, Developing countries, Perinatal mortality, Asphyxia neonatorum, Hemorrhage

## Abstract

**Background:**

There is a global increase in rates of Cesarean delivery (CD). A minor factor in this increase is a shift towards CD for breech presentation. The aim of this study was to analyze breech births by mode of delivery and investigate short-term fetal and maternal outcomes in a low-income setting.

**Methods:**

The study design was cross-sectional and the setting was Muhimbili National Hospital (MNH), Dar-es-Salaam, Tanzania. Subjects were drawn from a clinical database (1999–2010) using the following inclusion criteria: breech presentation, birth weight ≥ 2,500 g, single pregnancy, fetal heart sound at admission, and absence of pregnancy-related complication as indication for CD. Of 2,765 mothers who had a breech delivery, 1,655 met the inclusion criteria. Analyses were stratified by mode of delivery, taking into account also other birth characteristics. The outcome measures were perinatal death (stillbirths + in-hospital neonatal deaths) and moderate asphyxia. Maternal outcomes, such as death, hemorrhage, and length of hospital stay, were also described.

**Results:**

The CD rate for breech presentation increased from 28 % in 1999 to 78 % in 2010. Perinatal deaths were associated with vaginal delivery (VD) (adjusted odds ratio (aOR) 6.2; 95 % confidence interval (CI) 3.0–12.6) and referral (aOR 2.1; 95 % CI 1.1–3.9), but not with parity, birth weight, or delivery year. Overall perinatal mortality was 5.8 % and this did not decline, due to an increase in stillbirths among vaginal breech deliveries. Mothers with CD had more hemorrhage compared to those with VD. One mother died in association with CD, and one died in association with VD.

**Conclusion:**

A breech VD, compared to a breech CD, in this setting was associated with adverse perinatal outcome. However, despite a significant increase in CD rate, no overall improvement was observed due to an increase in stillbirths among VDs.

## Background

In 2000, the Term Breech Trial (TBT) concluded that planned Cesarean delivery (CD) (compared to planned vaginal birth) for breech presentation improved fetal outcome, though only modestly increasing maternal morbidity [1]. A systematic review in 2015 by Berhan et al. reports a substantial increase in elective CDs for breech presentation in high-income countries since 2000; at the same time, a two- to fivefold risk increase in perinatal mortality and morbidity has been reported with planned vaginal delivery (VD) [2, 3].

The TBT reports that, in settings with a low perinatal mortality rate (PMR) (≤ 20/1,000), the rate of perinatal mortality/serious morbidity was 0.4 % for planned CD and 5.3 % for planned vaginal delivery (VD), whereas in settings with a high PMR (> 20/1,000), in one low- and eight middle-income countries, the rate of perinatal mortality/serious morbidity was 2.9 % for planned CD and 4.4 % for VD [1]. These results challenge the TBT’s conclusions with respect to the effect of planned CD on perinatal outcome in a low-income setting, where CDs for breech presentation are increasing [3].

Maternal complications, such as blood transfusions, hysterectomy, and admission to the intensive care unit (ICU), as well as death, are estimated to be twice as common in CD as in VD in a low-income setting [4]. In low-resource settings, the maternal death rate for CD ranges between 0.1 and 1.9 % [3, 5, 6]. According to a study from the Netherlands, the overall maternal fatality rate since the TBT’s conclusions have been published has been 0.25 per 1,000 breech CD and 0.47 per 1,000 planned breech CD [7].

Vaginal delivery for breech presentation is still a recommended option where possible [2, 8, 9], especially in low-income settings where CD-associated maternal morbidity and mortality are a serious consideration [3, 6, 10]. The few observational studies of breech delivery in sub-Saharan African Hospitals report a wide range of short-term outcomes, reflecting resource constraints and policy differences [6, 11–13]. There is a lack of continuous statistical surveillance in many busy hospital obstetrics units in low-income settings. More investigation is needed to better understand the consequences of a policy shift in mode of delivery in low-income settings. To this end, we analyzed maternal and fetal outcomes in breech delivery by mode of delivery and birth characteristics, specifically intrapartum fetal deaths, asphyxia, early neonatal deaths, and maternal outcomes. Our study was conducted at a University Hospital in Tanzania, a low-income setting.

## Methods

This cross-sectional study was performed at a teaching and referral hospital, Muhimbili National Hospital (MNH), in Dar-es-Salaam, Tanzania. Recently, district hospitals in the region have been upgraded, resulting in fewer deliveries at MNH [14]. At MNH, the CD rate has increased, from 16 % in 1999 [15] to 49 % in 2011 [16]. Between 2000 and 2011, CDs for nulliparous and multiparous women increased by 131 and 171 %, respectively [16].

According to the recommendation from the Ministry of Health and Social Welfare, external cephalic version should be performed at 34–36 weeks in all persistent breech presentations if detected before labor. Breech presentation at admission to the delivery ward is diagnosed by external and internal examination. For primiparas, mode of delivery is based on the size of the fetus and the clinical assessment of the mother. All multiparas without previous CD undergo breech delivery assisted by a senior midwife or obstetrician. Fetal heart rate is monitored using a fetoscope/Doppler device. Mothers with uncomplicated VD are discharged early, usually 6 h after delivery. Indications for CD are a large baby, poor progress, fetal distress, a previous CD, a deformed pelvis, hydrocephalus, or umbilical cord prolapse. Antibiotics are prescribed for emergency CD.

In 1998, an obstetric database was created at MNH, an electronic registry to support research and quality development. To validate the data, a quality control program and manual checks of selected variables are run weekly [17].

**Fig. 1:**
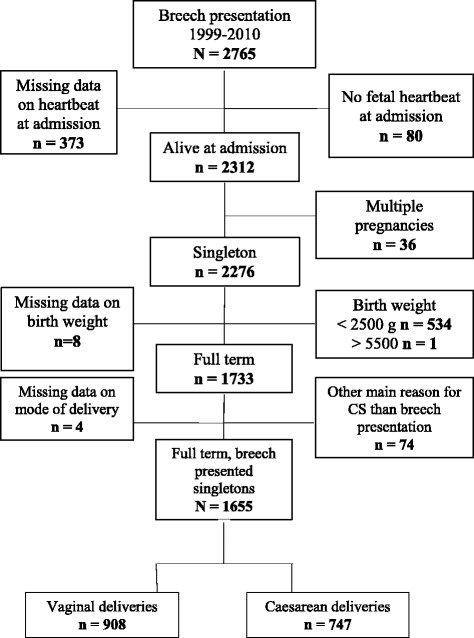
Flow chart. Inclusion and exclusion criteria for the study sample of breech deliveries at Muhimbili National Hospital (MNH), Dar-es-Salaam, Tanzania, 1999–2010

In this study, we selected a sample to investigate perinatal outcomes following breech presentation between 1999 and 2010 (Fig. 1). The primary selection from the database was drawn only from the first hierarchy variable giving the maternal diagnosis as “breech” (*N* = 2,765). Although breech presentation was also noted among the second and third maternal diagnostic variables, those subjects had mainly “twin pregnancy” and “hypertension” as first diagnosis. The inclusion criteria included delivery between 1999 and 2010, live fetus at admission, and delivery of a singleton baby with a birth weight (BW) ≥ 2,500 g. Exclusion criteria are described in Fig. 1. Mothers with conditions (*n* = 74) that were potential indications for CD (e.g., hypertension, eclampsia, antepartum hemorrhage, and abruptio placentae) were excluded.

The final sample consisted of 1,655 breech deliveries. The following patient characteristics were recorded: age, parity, mode of delivery, BW, referral (yes/no), insurance status, and year of delivery (stratified by period). By “referral” is meant whether the mothers had been transferred from a district hospital. Regarding “private insurance,” patients at MNH are either private or public. “Mode of delivery” was categorized into VD or CD. The option “emergency or elective CD” has been available since 2005, but was missing in 75 % of cases, with < 10 % of cases being categorized as “elective;” hence, this option was not used. The variable “mode of delivery” therefore contained information about whether the delivery was a CD, or a spontaneous or assisted breech VD, but only 4 % of VDs were coded into one category together with the assisted breech VDs.

The analysis was stratified by mode of delivery (VD/CD). The other independent variables, parity (primiparous/multiparous), BW (2.5–3.6 kg/3.7–4.7 kg), referral (yes/no), privately insured (yes/no), and year of delivery (1999–2004/2005–2006/2007–2010), were separately analyzed. Five outcome variables were recorded: (1) hemorrhage = blood loss ≥ 1,000 ml; (2) moderate asphyxia = Apgar score < 4 at 5 min (code P21.1B, International Classification of Diseases and Related Health, 10^th^ revision (ICD10); (3) stillbirth = fetuses with presence of heartbeat at admission, but no sign of vitality at birth; (4) in-hospital neonatal death = death before discharge from MNH; and (5) perinatal death = fetuses with presence of heartbeat at admission, but no sign of vitality at birth, or with death before discharge from MNH.

Data were analyzed in IBM SPSS statistics version 23 (IBM Inc., Armonk, NY, USA) using Pearson’s chi-square test, Fisher’s exact test, and Mantel-Haenszel chi-square test for linear trends. *P*-values < 0.05 were considered statistically significant. Crude odds ratios (ORs) and 95 % confidence intervals (95 % CIs) were calculated. In the final model of the multivariate analysis by logistic regression, relevant exposure variables were included when estimating adjusted odds ratios (aORs).

## Results

**Fig. 2:**
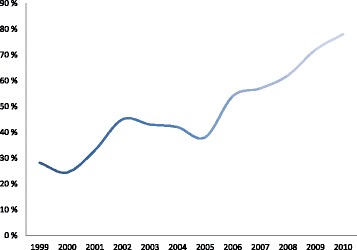
Cesarean delivery (CD) rate for breech presentation at Muhimbili National Hospital (MNH), Dar-es-Salaam, Tanzania, 1999–2010

In our sample of 1,655 women with breech presentation, 908 (54.8 %) had a VD and 747 (45.1 %) had a CD (Fig. 1). The rate of CDs for breech increased from 28 % in 1999 to 78 % in 2010 (Fig. 2).

The percentage alive at discharge was 97.5 % for the CD group and 91.5 % for the VD group. In the CD group, rates of stillbirths, in-hospital neonatal deaths, and asphyxia were 1.6, 0.9, and 0.7 %, respectively; in the VD group, corresponding rates were 3.9, 4.6, and 3.3 %. All differences were statistically significant (Table 1). Every eleventh vaginally delivered infant died, compared to every 39th infant delivered by Cesarean section. Cause of death was registered in approximately half of the infants who did not survive. The vast majority in both groups (84 %) died from birth asphyxia. Both the CD and the VD groups had one death due to meconium aspiration. In the VD group, there were two deaths from severe hypoxic ischemic encephalopathy (HIE) and three from “prematurity-related complications,” although these cases met our inclusion criteria.

**Table 1:** Birth outcomes of vaginal delivery (VD) versus Cesarean delivery (CD) for breech presentation, at Muhimbili National Hospital (MNH), Dar-es-Salaam, Tanzania, 1999–2010 (*N* = 1,655)

				VD		CD		*P*-value
				*n*	%	*n*	%	
All			Alive at discharge	831	91.5	728	97.5	<0.0001
			Stillborn	35	3.9	12	1.6	0.007
			In-hospital neonatal death	42	4.6	7	0.9	<0.0001
			Apgar score < 4^5^	29	3.3	5	0.7	<0.0001
	Parity	Primipara	Alive at discharge	274	90.1	379	97.4	
			Stillborn	12	3.9	5	1.3	0.018
			In-hospital neonatal death	18	6.0	5	1.3	0.000
			Apgar score < 4^5^	25	8.2	10	2.6	0.001
		Multipara	Alive at discharge	557	92.2	349	97.5	
			Stillborn	23	2.0	7	2	0.000
			In-hospital neonatal death	24	4.0	2	0.5	0.001
			Apgar score < 4^5^	38	8.2	7	2.0	0.009
	Birth weight	2.5–3.6 kg	Alive at discharge	772	91.6	616	97.8	
			Stillborn	32	3.8	8	1.3	0.002
			In-hospital neonatal death	39	4.6	6	1.0	0.000
			Apgar score < 4^5^	59	13.8	6	5.1	0.018
		3.7–4.7 kg	Alive at discharge	59	90.8	112	95.7	
			Stillborn	3	4.6	4	3.4	0.650
			In-hospital neonatal death	3	4.6	1	0.9	0.094
			Apgar score < 4^5^	4	6.2	9	3.4	0.001
	Referral	No	Alive at discharge	745	92.7	577	98.1	
			Stillborn	25	3.1	6	1.0	0.007
			In-hospital neonatal death	34	4.2	5	0.9	0.000
			Apgar score < 4^5^	23	3.0	3	0.5	0.000
		Yes	Alive at discharge	86	82.7	151	95.0	
			Stillborn	10	9.6	6	3.7	0.036
			In-hospital neonatal death	8	7.7	2	1.3	0.005
			Apgar score < 4^5^	6	6.4	2	1.3	0.029
	Privately insured	Yes	Alive at discharge	12	100	91	98.9	
			Stillborn	0	–	1	1.1	
			Neonatal death	0	–	0	–	
			Apgar score < 4^5^	0	–	0	–	
		No	Alive at discharge	247	85.5	343	96.9	
			Stillborn	20	6.9	9	2.5	0.004
			In-hospital neonatal death	22	7.6	2	0.6	0.000
			Apgar score < 4^5^	2	0.6	15	5.6	0.000

“Stillborn” is defined as fetuses with heart sound at admission, but no sign of vitality at birth; “in-hospital neonatal deaths” is defined as live births, with death taking place before discharge from hospital; “moderate asphyxia” is defined as Apgar score <4 at 5 min. Pearson’s chi-square test was used to analyze the data

Cesarean delivery was associated with fewer stillbirths, in-hospital neonatal deaths, and moderate asphyxia, irrespective of parity, presence or absence of referral, BW (2.5–3.6 kg), and insurance status. For infants with BW between 3.7 and 4.7 kg, CD was associated with less moderate asphyxia (Table 1). Regarding year of delivery, there was an increasing trend, over time, of stillbirths among VD breech babies of women not referred, but not of in-hospital neonatal deaths. The low survival of VD breech babies referred to MNH did not change over time. For breech babies delivered by Cesarean section, there were no changes over time in survival with respect to whether the mother had been referred or not (Table 2).

**Table 2:** Birth outcomes of vaginal delivery (VD) versus Cesarean delivery (CD) for breech presentation, by year of delivery, at Muhimbili National Hospital (MNH), Dar-es-Salaam, Tanzania, 1999–2010 (*N* = 1,655)

		1999–2004		2005–2006		2007–2010		
Mode of delivery		*n*	%	*n*	%	*n*	%	*P*-value
VD								
Not referred								
	Alive at discharge	580	94.0	73	90.2	92	86.8	0.007
	Stillborn	13	2.1	4	4.9	8	7.5	0.002
	In-hospital neonatal death	24	3.9	4	4.9	6	5.7	0.099
Referred								
	Alive at discharge	48	88.9	10	71.4	28	77.8	0.1999
	Stillborn	3	5.6	2	14.3	5	13.9	0.221
	In-hospital neonatal death	3	5.6	1	14.3	3	8.3	0.662
CD								
Not referred								
	Alive at discharge	287	98.3	62	96.9	228	77.8	0.903
	Stillborn	1	0.3	2	3.1	3	1.3	0.371
	In-hospital neonatal death	4	1.4	0	0	1	0.4	0.300
Referred								
	Alive at discharge	52	96.3	11	78.6	88	96.7	0.595
	Stillborn	1	1.9	2	14.3	3	3.3	0.983
	Early neonatal death	1	1.9	1	7.1	0	0	0.136
All VDs								
	Alive at discharge	628	93.6	83	87.4	120	84.5	0.000
	Stillborn	16	2.4	6	6.3	13	9.2	0.000
	In-hospital neonatal death	27	4.0	6	6.3	9	6.3	0.141
All CDs								
	Alive at discharge	339	98.0	73	93.6	316	97.8	0.0002
	Stillborn	2	0.6	4	5.1	6	1.9	0.242
	In-hospital neonatal death	5	1.4	1	1.3	1	0.3	0.089
Total								
	Alive at discharge	967	95.1	156	90.2	436	93.8	0.212
	Stillborn	18	1.8	10	5.8	19	4.1	0.008
	In-hospital neonatal death	32	3.1	7	4.0	10	2.2	0.354

“Stillborn” is defined as fetuses with heart sound at admission, but no sign of vitality at birth; “in-hospital neonatal deaths” is defined as live births, with death taking place before discharge from hospital. Analyses were performed using Mantel-Haenszel chi-square test for linear trends

Adjusted OR for perinatal death for VD breech was 6.2 (95 % CI 3.0–12.6) and for referral, 2.05 (95 % CI 1.09–3.86). Neither parity, nor BW, insurance status, or year of delivery was associated with perinatal death with respect to mode of delivery (Table 3).

**Table 3:** Risk of moderate asphyxia (defined as 5-min Apgar score < 4) and perinatal death (defined as fetuses with heart sound at admission, but no sign of vitality at birth, or as live birth, but with death before discharge from Hospital) for breech births, by mode of delivery, parity, birth weight, and referral, at Muhimbili National Hospital (MNH), Dar-es-Salaam, Tanzania, 1999–2010 (*N* = 1,655)

		OR	95 % CI	aOR	95 % CI
Mode of delivery					
CD	Perinatal death	1		1	
VD		3.55	2.13–5.92	6.18	3.04–12.6
CD	Apgar score < 4^5^	1		1	
VD		5.02	1.93–13.03	5.97	2.18–16.3
Parity					
Multipara	Perinatal death	1		1	
Primipara		0.99	0.65–1.50	1.25	0.68–2.30
Multipara	Apgar score < 4^5^	1		1	
Primipara		1.77	0.89–3.51	2.22	1.10–4.48
Birth weight					
2.5–3.6 kg	Perinatal death	1		1	
3.7–4.7 kg		1.05	0.55–2.00	1.78	0.74–4.28
2.5–3.6 kg	Apgar score <4^5^	1		1	
3.7–4.7 kg		0.24	0.03–1.79	0.38	0.05–2.82
Referral					
No	Perinatal death	1		1	
Yes		2.07	1.29–3.32	2.05	1.09–3.86
No	Apgar score < 4^5^	1		1	
Yes		1.72	0.77–3.84	2.24	0.96–2.82
Insurance					
Yes	Perinatal death	1		1	
No		9.25	1.27–67.5	3.99	0.52–30.41
Time period (delivery year)					
1999–2004	Perinatal death	1		1	
2005–2006		2.11	1.19–3.75	1.49	0.61–3.67
2007–2010		1.29	0.80–2.06	1.26	0.54–2.94
1999–2004	Apgar score < 4^5^	1		1	
2005–2006		1.70	0.68–4.25	1.94	0.76–4.94
2007–2010		0.61	0.24–1.50	0.87	0.33–2.31

Crude odds ratios (ORs), adjusted odds ratios (aORs), and 95 % confidence intervals (CIs) are shown. *CD* cesarean delivery, *VD* vaginal delivery

Hemorrhage was more common for the CD (7.2 %) than the VD group (1.0 %) (*p* = 0.0001). There were two maternal deaths: in the CD group, one mother died from “anesthetic complications;” in the VD group, one mother died from a ruptured uterus.

## Discussion

This study is one of the few studies to analyze, in a large sample, the difference in outcome between breech VD and breech CD in a low-income country. During the study period, there was an almost threefold increase in breech CDs. The risks of perinatal death and moderate asphyxia were significantly higher among infants delivered vaginally; and for perinatal death, they were higher if the mother had been referred, irrespective of parity, BW, insurance status, and delivery year. Despite the increase in breech CDs, overall perinatal mortality in breech births did not decrease as there was an increase in stillbirths among vaginally delivered breech babies.

In agreement with earlier studies, the present study shows improved fetal outcome for breech fetuses, in terms of intrapartum deaths, early neonatal deaths, and asphyxia, when delivered by CD compared to VD [1, 2, 4, 8]. Results from other, similar settings do not, however, completely agree with our findings. Studies from Guinea and Nigeria found low Apgar scores to be more frequent among VD than CD for breech presentation [12, 13, 18] although the Guinean study showed no difference in PMR between the groups [18]. As in our study, a study from Zimbabwe demonstrated a significant reduction in PMR for breech presentation (OR 5.4, *p* < 0.001), but saw no correlation between changes in CD rate and PMR [6]. In 2006, a Nigerian study showed a significant reduction in PMR for infants of primigravidae with BW > 3,500 g when delivered by CD compared to VD [11].

The almost threefold increase in CD rate for breech presentation was not associated with an overall improvement in breech births or improved survival for breech-delivered infants. This is contrary to the TBT study and other studies in Western settings [1, 8, 19, 20]. One explanation, at least a partial explanation, for this difference might be selection bias, as there was a gradual improvement in maternity care in Dar-es-Salaam as the surrounding district hospitals improved [17]. Muhimbili National Hospital had a 40 % decrease in deliveries between 2000 and 2002 and between 2009 and 2011, which was concomitant to an increase in referral cases, from 7 to 28 % [16]. The higher proportion of referred patients also includes patients with breech presentation in labor, and they had worse outcome, irrespective of mode of delivery. Another reason for this difference could have been reduced staff skills in assisted breech delivery, as, in our sample, the number of vaginal breech deliveries decreased from three per week to one every 2 weeks [3]. Van Roosmalen and Meguid highlight that settings that increasingly use CD may not have trained staff with the skills to assist vaginal breech delivery, and that this staff will need skills training in this area [3]. Hannah et al. found that planned CD for breech presentation did not reduce serious morbidity in newborns in high-PMR countries as much as in low-PMR countries. They recognized the possibility of the caregivers being more experienced in breech deliveries in the low-PMR countries, which traditionally have low CD rates [1].

It is unclear how selection for the two different modes of delivery in this study was carried out. Women who delivered vaginally might have represented good candidates for a trial of labor, although facilities for such assessments are not the same at MNH as in a facility in a high-income country. Lead time from decision to operate can in this low-income setting be extended by several hours [21], meaning that VDs could represent a group of most urgent cases that did not make the necessary conversion to CD. This might be one explanation for the clustering of VD stillbirths, indicating the difficult conditions prevailing in this setting, especially as the study sample comprised of presumed intrapartal deaths. Birth asphyxia was the cause of neonatal death for all CDs and nine out of ten VDs.

Mothers with private insurance had excellent reported perinatal outcomes, which might indicate socioeconomic disparities and/or different quality of care. However, they constituted only 6 % of the sample, and did not influence the overall results.

As expected, women with CD suffered from hemorrhage more often, and one out of 25 had significant blood loss. One maternal death was caused by anesthetic complications. We had no information about postoperative complications such as rupture of the wound, infection, thromboembolism, or readmissions. Lack of registrated postpartum complications is a major weakness of the study as it makes it difficult to properly evaluate risks connected to CD. In high-income settings, 17 % of CDs may be complicated by maternal infectious morbidity [22]; the TBT study found a postpartum systemic infection rate of 1.5 % and a wound infection rate of 1.5 % for CD [1]. However, Litorp et al. report, in a study conducted at MNH in 2012 and published in 2014, an overall CD complication risk per 1,000 operations for maternal death of 1.0 (0.1–3.6) and for life-threatening complications of 6.0 (3.1–10) [5]. Based on these figures, two to four cases of life-threatening complications among CDs in this study could have occurred in this sample [5].

Consideration of complications is important when assessing indications for CD in developing countries and these should be weighed against the benefits of operation. The risk of uterine rupture is increased by up to 35 times for women in labor who have had a previous CD, compared to no history of CD [23]. Placenta accreta is three times more common in women with previous CD [24]. However, neither short-term complications after discharge nor long-term outcome could be addressed in this study.

In a cost-effectiveness analysis of strategies for maternal and neonatal health in developing countries, CD performed for breech presentation, obstructed labor, and fetal distress in conjunction with emergency neonatal care was estimated to be cost-effective in East African and South East Asian countries [25]. A cost analysis of hospital deliveries in low-PMR countries that was conducted in 2006 reports that, with regard to breech presentation, CD was less expensive compared to VD (US$7,165 versus US$8,042) [26].

Concerns about the increasing CD rates in low-income countries have been raised [4, 6, 10, 16, 27], although breech presentation represents a small percentage (1.7 %) of indications for CD at MNH and although breech benefits from CD [1, 4, 11, 12, 25]. Vaginal delivery of breech presentation still remains an option and the systematic review by Berhan et al. supports “the practice of individualised decision-making on the route of delivery” [2].

One strength of this study is its unique database: All the deliveries were performed in a busy University Hospital in a low-income setting. However, the database has limitations. There may have been underreporting of breech deliveries. Also, it was not possible to determine whether the decision to perform CD was made before or during labor; this could not be analyzed because the variable “elective/emergency” was missing in 75 % of cases. Most of the decisions to perform CDs were probably made during labor, which may explain the high mortality and morbidity rate related to CD in this setting.

We were unable to describe early neonatal mortality. Discharge is normally 6 h after a VD and 3 days after a CD, so the rate of neonatal deaths may have been underestimated, especially among the VD cases.

## Conclusion

In conclusion, this study in a Tanzanian population shows that CD for breech presentation was associated with improved perinatal outcome, but that there was no overall improvement in perinatal outcome for breech presentation. Indications for CD should always be carefully evaluated, but this is especially important in resource-poor settings such as sub-Saharan Africa. Skills training for assisted vaginal breech delivery needs to be strengthen and maintained.

### Key message

Cesarean delivery for breech in a Tanzanian University Hospital was associated with improved perinatal outcome. Overall mortality was, however, unchanged due to an increase in stillbirth among vaginal deliveries.
